# Pelvic lymphangioleiomyomatosis treated successfully with everolimus: Two case reports with literature review

**DOI:** 10.1097/MD.0000000000004562

**Published:** 2017-03-10

**Authors:** Sharjil Wahid, Ping Chia Chiang, Hao Lun Luo, Shun-Chen Huang, Eing-Mei Tsai, Po Hui Chiang

**Affiliations:** a Department of Urology, Kaohsiung Chang Gung Memorial Hospital and Chang Gung University College of Medicine; b Graduate Institute of Medicine, College of Medicine, Kaohsiung Medical University; c Department of Pathology, Kaohsiung Chang Gung Memorial Hospital and Chang Gung University College of Medicine; d Department of Obstetrics and Gynecology, Kaohsiung Medical University Hospital, Kaohsiung Medical University; e Graduate Institute of Medicine, Center of Excellence for Environmental Medicine, Kaohsiung Medical University, Kaohsiung, Taiwan; f College of Physicians and Surgeons, Pakistan; g School of Medicine, Kaohsiung Medical University, Kaohsiung, Taiwan.

**Keywords:** angiomyolipoma, everolimus, lymphangioleiomyomatosis, mammalian target of rapamycin (mTOR) inhibitor, tuberous sclerosis

## Abstract

**Background:**

Lymphangioleiomyomatosis (LAM) is a rare disease affecting young women caused by abnormal proliferation of smooth muscle-like cells (LAM cells) in the lungs and extrapulmonary sites (extrapulmonary LAM). The objective of this case series is to demonstrate marked regression in 2 cases of retroperitoneal LAM after treatment with everolimus, an mTOR inhibitor.

**Methods:**

We enrolled 2 cases with large volume, extrapulmonary pelvic LAM, and evaluated them with contrast-enhanced abdominal computed tomographic (CT) scans at presentation and serially during treatment with everolimus. Results were objectively quantified using the Response Evaluation Criteria in Solid Tumors, RECIST, Version 1.1.

**Results:**

After 12 to 18 months of treatment with everolimus, both patients showed substantial reduction in the volume of their tumors. The first had about 50% regression of the pelvic LAM and renal angiomyolipoma (AML). The second patient had extensive abdomino-pelvic LAM which after treatment showed complete remission. Both patients have not demonstrated disease progression after nearly 4 and 2 years of follow-up, respectively.

**Conclusions:**

This case series demonstrates the enormous value of mTOR inhibitors (specifically everolimus) in the management of extrapulmonary pelvic LAM, of which there is no effective treatment currently available.

## 1 Introduction

Lymphangioleiomyomatosis (LAM) is a rare disease affecting young women of child-bearing ages.^[1,2]^ It occurs both sporadically (sLAM) and in association with tuberous sclerosis complex (TSC-LAM). sLAM has a prevalence of 1:1,000,000, but is much commoner in TSC patients. About a third of all women with TSC have co-existing LAM.^[3,4]^ LAM arises from mutations in the TSC genes (TSC 1 and 2), which inactivates them. These genes encode the proteins hamartin and tuberin that combine to form a complex (TSC1–TSC2 complex). This complex then inhibits and regulates the mammalian target of rapamycin (mTOR) protein, via the mTORC1 pathway. This is a key regulator of cell proliferation and lymphangiogenesis. In LAM, the absence of the TSC1–TSC2 complex leads to uncontrolled activation of mTOR.^[5]^ Abnormal proliferation of smooth muscle-like cells (LAM cells) results, leading to development of tumors (mostly hamartomas) in various sites.^[6,7]^ Although these mainly form in the lungs, extrapulmonary involvement also occurs, typically in the kidneys, retroperitoneum, and pelvic regions.^[8,9]^ Cysts form in the lungs causing destruction of pulmonary architecture, whereas solid tumors develop in kidneys (angiomyolipomas) and retroperitoneal and abdomino-pelvic sites (lymphangioleiomyomas). Lymphatic obstruction causes chylothorax and chylous ascites.^[10]^ These tumors have characteristics of low grade neoplasms, including the potential to metastasize.^[11]^

Pharmacologic inhibitors of mTOR, such as everolimus and sirolimus, directly inhibit T-lymphocyte proliferation. Although there is ample evidence that mTOR inhibitors are effective in the treatment of pulmonary and renal LAM, there are limited studies which prove their efficacy in pelvic LAM. Currently, there is no effective treatment for extrapulmonary LAM.^[12–14]^ We herein present 2 cases of extrapulmonary pelvic LAM which were successfully treated with everolimus (Afinitor—Novartis Pharmaceuticals), an mTOR inhibitor.

### 1.1 Case 1

**Figure 1:**
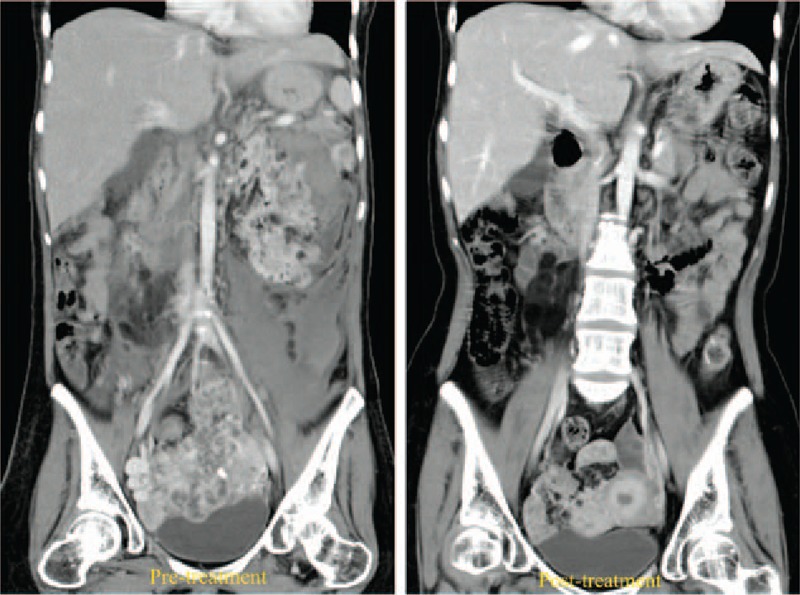
Contrast-enhanced abdominal CT images of the first patient before and after 4 months of everolimus 10 mg/ day treatment. Pretreatment image shows ruptured left AML and a large pelvic mass. Post-treatment, significant regression was seen in both renal and pelvic tumors. AML = angiomyolipomas, CT = computed tomography.

A 37-year-old Asian lady presented to the emergency department with sudden onset of back pain and fainting. She was an epileptic, had mental retardation, and a history of progressive low abdominal fullness since a year. Physical examination revealed facial angiofibromatosis, a tender palpable mass in the lower abdomen and ecchymosis over the flank. Relevant routine blood laboratory investigations were (values [reference range]): white blood count 9.8 × 10^9^ /L (4–11), hemoglobin 6.1 g/dL (12–15), creatinine 1.59 mg/dL (0.8–1.3). A contrast-enhanced abdominal CT revealed multiple bilateral renal angiomyolipoma (AML), with confined retroperitoneal hemorrhage around the left kidney. An extensive pelvic tumor, 153.12 × 100.03 mm in the transverse plane, was also seen almost filling the pelvic cavity, resulting in compression of the urinary bladder, uterus, rectum, and small bowel. Genetic studies revealed TSC-2 gene mutation. A diagnosis of tuberous sclerosis (TSC) associated with bilateral AML and pelvic LAM was made and supportive care was instituted. Everolimus 10 mg/day was prescribed after informed consent. She made an uneventful recovery without surgery. Follow-up CT scans showed marked regression of the bilateral AML and pelvic LAM (Fig. 1). We used the Response Evaluation Criteria in Solid Tumors, RECIST, Version 1.1, to measure tumor response in an objective manner, in both patients. The largest pelvic tumor diameter was measured serially and the RECIST calculator showed that the pelvic mass (target lesion) shrunk to 105.72 mm within 4 months (–30.72%) and to 74.64 mm at 12 months (–50.98%). There was also notable regression of the renal AML (nontarget lesions) bilaterally. This amounted to a partial response. Adverse effects of the drug were explained in detail, and sought at follow-up sessions, but none were found except mild stomatitis (Table 1). No dose adjustment was necessary. Her epileptic seizures were also well controlled by the drug.

**Table 1:**
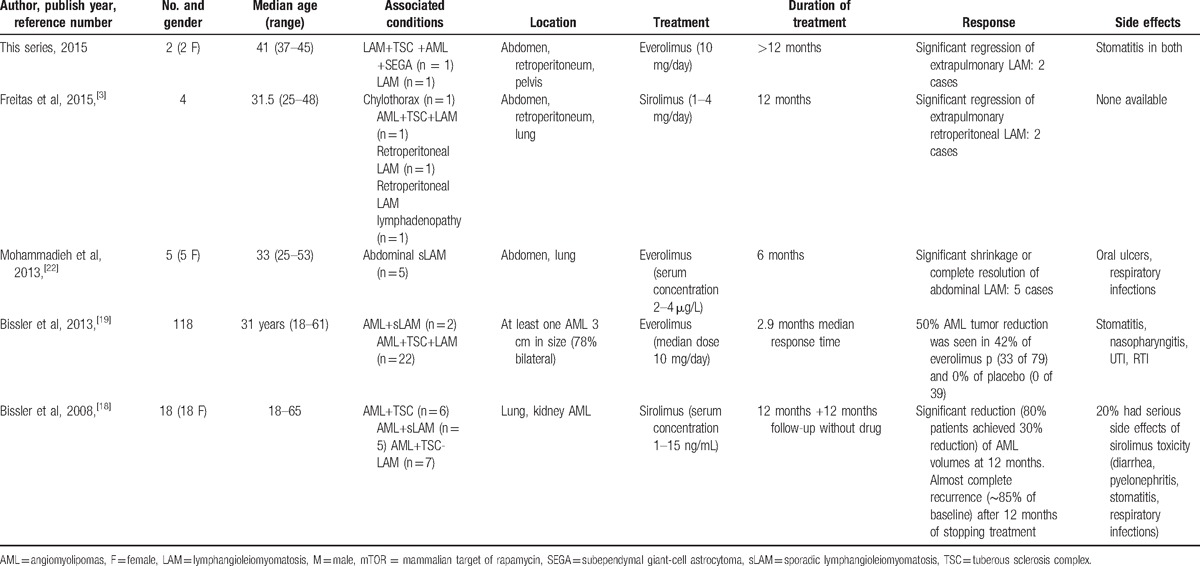
Comparison of patient data, treatment, and results of our study with other related published studies regarding use of mTOR inhibitors in extrapulmonary LAM.

### 1.2 Case 2

**Figure 2:**
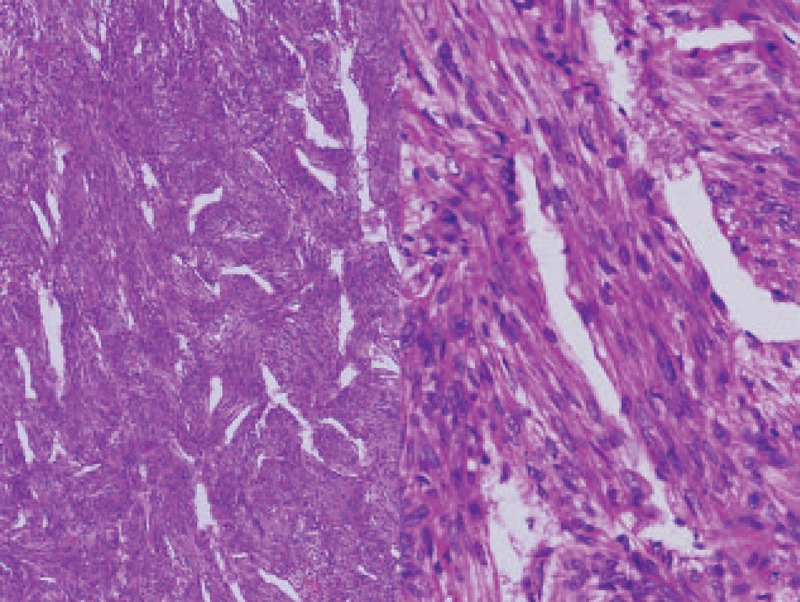
Hematoxylin and Eosin (H&E), at ×40 and ×200 magnification. Microscopically, the tumor was composed of fascicles of spindle cells, with intervening vascular channels. H&E = hematoxylin and eosin.

**Figure 3:**
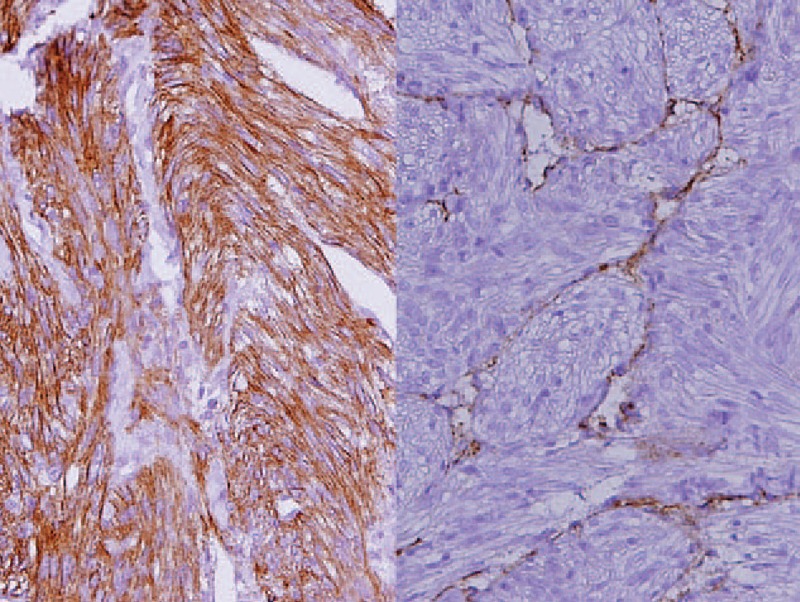
Immunohistochemical studies. Muscle-specific actin (HHF35) ×200 (left) showed that the spindle cells were smooth muscle cells. Lymphatic endothelial marker (D2–40) ×200 (right) confirmed that the endothelium was of lymphatic endothelium.

**Figure 4:**
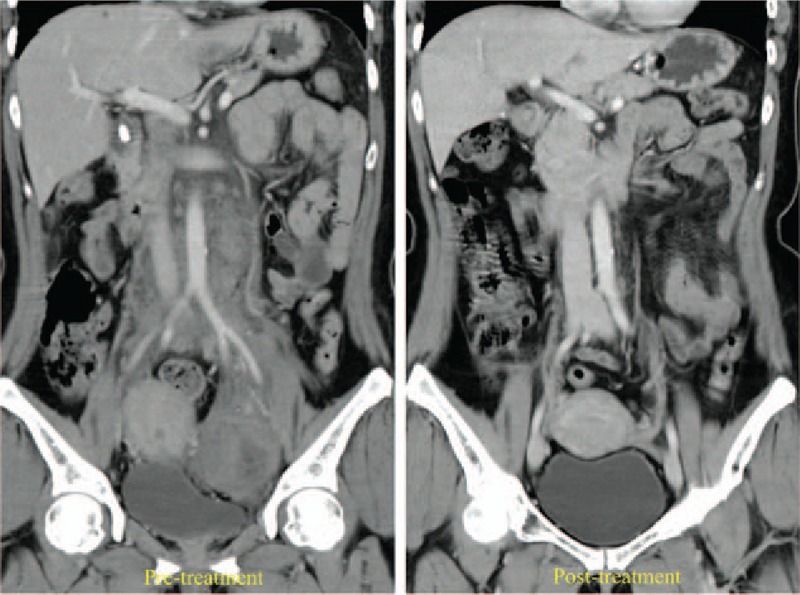
Contrast-enhanced abdominal CT images of the second patient before and after 3 months of everolimus treatment. The pretreatment tumor encased the inferior vena cava and aorta and extended into the pelvis. Complete remission was achieved at 18 months with everolimus 10 mg daily. CT = computed tomography.

A 45-year old Asian lady presented to the out-patient clinic with flank pain and low abdominal fullness for a year. Physical examination was unremarkable and routine blood laboratory investigations were (values [reference range]): white blood count 10.1 × 10^9^ /L (4–11), hemoglobin 14.9 g/dL (12–15), creatinine 0.7 mg/dL (0.8–1.3), Alanine aminotransferase 16 (5–30 U/L). Contrast-enhanced abdominal CT revealed a retroperitoneal mass, 85.12 × 33.94 mm, encasing the inferior vena cava, aorta and the left ureter from just below the renal vessels bifurcation to extend down into the pelvis on the left side causing displacement of the urinary bladder. The pelvic part of the tumor, 68.77 mm in longest diameter, was causing pressure compression to the uterus, rectum, and small bowel. No lesions were observed in the kidneys. An open tumor biopsy revealed that the tumor was composed of fascicles of spindle cells with intervening vascular channels and was confirmed as pelvic LAM on immunohistochemical studies (Figs. 2 and 3). Genetic studies revealed no TSC-1 or 2 gene mutation. Hence, a diagnosis of nontuberous sclerosis-associated abdomino-pelvic LAM (sLAM) was made. The patient was started on Everolimus 10 mg/day after informed consent. She consequently became symptom free with minimal adverse effects, which did not require dose modification (Table 1). Follow-up abdominal CT after 3 months found significant reduction of the pelvic tumor (target lesion) to 33.12 mm (–52.17%) and complete disappearance (–100%) at 18 months (Fig. 4). The retroperitoneal mass (nontarget lesion) showed complete regression at 3 months. Thus, there was a partial response at 3 months and a complete response at 18 months.

## 2 Discussion

Lymphangioleiomyomatosis (LAM) is a rare genetic disease of young women, in which tumors and cysts form in the lungs, kidneys (AML), and pelvic regions (pelvic LAM), causing pressure effects and symptoms. These tumors behave as low-grade neoplasms and can metastasize.^[11]^ Perhaps owing to the rarity of the condition, there is no effective treatment for thousands of LAM patients worldwide, as of current.^[14]^

To look for a cure, researchers turned to its aetio-pathology. As the disease occurred in reproductive-age women, some believed that estrogens may play a part in pathogenesis.^[6]^ LAM cells express estrogen-α and progesterone receptors (ER, PR) that activate certain pathways which stimulate cell growth. The disease was also observed to often worsen during pregnancy or with exogenous estrogens and contraceptive pills.^[15]^ Hence, progesterone, gonadotrophin-releasing-hormone (GnRH) analogs and oophorectomy have been tried as treatments, though only progesterone and oophorectomy seemed to offer a benefit in selected patients.^[16]^

It was known that LAM was somehow associated with tuberous sclerosis complex (TSC) since they often show up together. The TSC proteins were found to regulate the mTOR pathway, and activation of mTOR lead to uncontrolled cell growth and progression of LAM. This suggested that mTOR inhibitors, such as everolimus and sirolimus, may be a potential molecular therapy for this condition. These were shown to have beneficial effects on different manifestations of TSC including AML, LAM, and SEGA (subependymal giant-cell astrocytoma).^[12,14,17]^ In our case series, the first patient had TSC, whereas the second did not.

Bissler et al^[18]^ in 2008 conducted a 24-month clinical trial to study the effects of sirolimus on TSC and LAM manifestations (pulmonary cysts and renal AML). They demonstrated that AML lesions regressed and lung function improved with sirolimus therapy. In 2013, Bissler et al^[19]^ undertook a placebo-controlled trial with another mTOR inhibitor, everolimus, and reported that 50% AML shrinkage was achieved in 42% of patients who received everolimus and 0% in the placebo arm. Similar results were reported by Davies et al,^[20]^ who showed 30% AML tumor reduction in 50% of patients with sirolimus. However, the effects on pelvic LAM were not the focus of these studies, as is ours.

Krueger et al^[21]^ studied the effects of everolimus on refractory epilepsy in TSC patients. Patients with confirmed TSC and refractory epilepsy were treated with everolimus for 3 months. Overall seizures were reduced in 17 out of 20 patients (median 73%). Our first patient also demonstrated good seizure control with everolimus.

The treatment of extrapulmonary LAM is challenging. Although mTOR inhibitors are effective in the treatment of pulmonary and renal LAM, (US FDA approved), there are limited studies which demonstrate their efficacy in extrapulmonary LAM, as in our patients.^[12,13,22]^ To our knowledge, only 2 published articles have reported the effects of mTOR inhibitors on extrapulmonary LAM. Mohammadieh^[22]^ conducted an open-label treatment with Everolimus on 5 women with sporadic LAM (like our second patient) in Australia. LAM tumors in all 5 patients showed shrinkage with everolimus treatment for 6 months. The other case series, by Freitas et al^[3]^ in 2015, reported improvement in all 4 extrapulmonary LAM patients, after treatment with sirolimus for 12 months. This included reduction of renal AML, abdominal LAM, and lymph node mass. These 2 case series, albeit small in size, point to the benefits of using mTOR inhibitors in patients with extrapulmonary LAM.

In our case series, we tabulated our findings against other series and studies mentioned (Table 1). We had 2 patients with extra-pulmonary pelvic LAM, 1 TSC-associated and the other sLAM. Both patients had extensive pelvic tumors which were causing compression effects. We have focused on pelvic LAM in case series: to describe our experience of effects of everolimus on pelvic LAM tumors. Our patients were fully counseled regarding their disease and the drug everolimus, and gave informed consent to treatment. Both patients were started on everolimus 10 mg/day. Contrast-enhanced abdominal CT scans were serially performed and the Response Evaluation Criteria in Solid Tumors, RECIST, Version 1.1, was employed to objectively determine the effect, if any, of everolimus on pelvic LAM.^[23]^ The results were dramatic in our 2 patients. The first patient showed a 30.7% reduction of both pelvic LAM and renal AML at 4 months, which increased to 50.9% at 12 months. She also had stabilization of her epilepsy, although it is not clear whether that was as a result of everolimus or her antiepileptic medication or probably both. The second patient demonstrated an even better response, with a 52.1% decrease in pelvic tumor at 3 months, and complete remission at 18 months. They were advised to continue the treatment, in line with the findings of other studies (MILES trial, Bissler et al).^[12,18,22]^ At 4- and 2-year follow-up, respectively, both patients show sustained regression of the tumors.

There is debate on whether unidimensional measurements of the current RECIST guidelines adequately quantifies tumor burden. It is thought that volumetric tumor assessment by new CT/ MRI 3D software programs may be more reliable indicators of tumor change, though this has yet to be proved.^[24]^ In our patients, volumetric assessment may show a greater response than that described; however, even as it is, the results are impressive.

## 3 Conclusion

LAM is a rare genetic disease of women which mostly affects the lungs. Extrapulmonary involvement is very rare, and currently, its treatment has been ineffective. Although the efficacy of mTOR inhibitors in treating pulmonary and renal LAM is well established, very few studies have demonstrated their use in extrapulmonary abdomino-pelvic LAM tumors. The case series we present is of 2 patients with pelvic LAM, who were successfully treated with everolimus. The tumors showed dramatic volume reduction and symptom control and have not showed progression. Our case series add to the evidence that everolimus is a promising treatment in the management of this disease. However, the database is small, and more research is required to establish the optimal dose and duration of treatment and to document long-term beneficial effects.
